# Aberration of miRNAs Expression in Leukocytes from Sporadic Amyotrophic Lateral Sclerosis

**DOI:** 10.3389/fnmol.2016.00069

**Published:** 2016-08-17

**Authors:** YongPing Chen, QianQian Wei, XuePing Chen, ChunYu Li, Bei Cao, RuWei Ou, Shinji Hadano, Hui-Fang Shang

**Affiliations:** ^1^Department of Neurology, West China Hospital, Sichuan UniversityChengdu, China; ^2^Department of Molecular Life Sciences, Tokai University School of MedicineIsehara, Japan; ^3^The Institute of Medical Sciences, Tokai UniversityIsehara, Japan; ^4^Research Center for Brain and Nervous Diseases, Tokai University Graduate School of MedicineIsehara, Japan

**Keywords:** amyotrophic lateral sclerosis, miRNAs, microarray, biomarker, pathway, hsa-miR-183

## Abstract

**Background:** Accumulating evidence indicates that miRNAs play an important role in the development of amyotrophic lateral sclerosis (ALS). Most of previous studies on miRNA dysregulation in ALS focused on the alterative expression in ALS animal model or in limited samples from European patients with ALS. In the present study, the miRNA expression profiles were investigated in Chinese ALS patients to explore leukocytes miRNAs as a potential biomarker for the diagnosis of ALS.

**Methods:** We analyzed the expression profiles of 1733 human mature miRNAs using microarray technology in leukocytes obtained from 5 patients with sporadic ALS (SALS) and 5 healthy controls. An independent group of 83 SALS patients, 24 Parkinson's disease (PD) patients and 61 controls was used for validation by real-time polymerase chain reaction assay. Area under the receiver operating characteristic curve (AUC) was used to evaluate diagnostic accuracy. In addition, target genes and signaling information of validated differential expression miRNAs were predicted using Bioinformatics.

**Results:** Eleven miRNAs, including four over-expressed and seven under-expressed miRNAs detected in SALS patients compared to healthy controls were selected for validation. Four under-expressed microRNAs, including hsa-miR-183, hsa-miR-193b, hsa-miR-451, and hsa-miR-3935, were confirmed in validation stage by comparison of 83 SALS patients and 61 HCs. Moreover, we identified a miRNA panel (hsa-miR-183, hsa-miR-193b, hsa-miR-451, and hsa-miR-3935) having a high diagnostic accuracy of SALS (AUC 0.857 for the validation group). However, only hsa-miR-183 was significantly lower in SALS patients than that in PD patients and in HCs, while no differences were found between PD patients and HCs. By bioinformatics analysis, we obtained a large number of target genes and signaling information that are linked to neurodegeneration.

**Conclusion:** This study provided evidence of abnormal miRNA expression patterns in the peripheral blood leukocytes of SALS patients. Leukocytes miRNAs provide a promising opportunity for detection of SALS. The specificity of under-expression of hsa-miR-183 in SALS needs to be confirmed by further miRNA studies on other neurodegenerative diseases.

## Introduction

Amyotrophic lateral sclerosis (ALS) is a fatal progressive neurodegenerative disease characterized by degeneration of motor neurons (MNs) located in the brain stem motor nuclei, spinal anterior horn neurons, and corticospinal tract, resulting in paralysis and death within 3–5 years from the onset of symptoms. In different population, the proportion of familial ALS (fALS) ranges from 1 to 19%, while the remains are sporadic ALS (SALS) with unknown cause (Rowland and Shneider, 2001). To date, hundreds of mutations in more than 20 genes have been implicated in the pathology of ALS, such as mutations in superoxide dismutase (*SOD1*), TAR DNA-binding protein 43 (*TARDBP*), fusion (*FUS*), and a hexanucleotide expansion on chromosome 9 in open reading frame 72 (*C9ORF72*). However, these causative genes explain only two-thirds of fALS patients and about 11% of SALS cases in Caucasian populations (Renton et al., 2014).

Multiple mechanisms, including glutamate-mediated excite-toxicity effects, abnormal astrocyte and microglial activation, deficiency of neurotrophic factor secretion, protein misfolding, and aggregations, mitochondrial dysfunction, rupture in the axonal passage, destruction in calcium metabolism, changes in skeletal proteins account for the selective vulnerability of MNs, were implicated in the pathogenesis of ALS. However, the exact mechanism underlying MNs death in ALS is still unknown. Accumulating evidence indicates that dysregulation of RNA processing pathway plays an important role in ALS (Droppelmann et al., 2014).

RNA processing is a tightly regulated, highly complex pathway, which includes RNA transcription, pre-mRNA splicing, editing, transportation, translation, and degradation of RNA (Bolognani and Perrone-Bizzozero, 2008). Several identified causative genes of ALS are related to RNA metabolism, such as angiogenin (*ANG*) (Gao and Xu, 2008), *FUS* (Kwiatkowski et al., 2009), *TARDBP* gene (Strong et al., 2007). In addition, several ALS-associated proteins, such as ELP3, SETX, were reported to be involved in RNA processing (Strong, 2010). Furthermore, the recent discovery of miRNAs, which are small endogenous non-coding RNAs of 19–25 nucleotides functioned as inhibitor mRNA translation of target protein-coding genes or induce its degradation, has provided new insight into the regulation of gene expression. Functional studies indicate that miRNA plays a significant role in a broad range of cellular and developmental processes, such as stem cell maintenance, differentiation, development and energy metabolism (Bartel, 2009; Friedman et al., 2009).

Aberrant miRNAs expression has been implicated in several neurodegenerative diseases, including ALS (Borovecki et al., 2005; Hebert and De Strooper, 2009; Miñones-Moyano et al., 2011; Cloutier et al., 2015). Different miRNA expression profiles in ALS have been reported in several studies (Cloutier et al., 2015). Up-regulation of miR-206 can slow down the progression of ALS and promote regeneration of neuromuscular synapses, but this up-regulation was also identified in other diseases, such as schizophrenia (Hansen et al., 2007) and cerebral ischemia (Jeyaseelan et al., 2008), suggesting that up-regulation of miR-206 is not specific for ALS. In addition, the limitations of previous studies on miRNAs in ALS include small sample size (De Felice et al., 2012; Campos-Melo et al., 2013; Ishtiaq et al., 2014), the use of monogenic animal models (Butovsky et al., 2012; Freischmidt et al., 2013; Marcuzzo et al., 2014; Nolan et al., 2014), or human biopsy tissue from skeletal muscle (Russell et al., 2012), spinal cord (Campos-Melo et al., 2013; Ishtiaq et al., 2014) or frontal cortex (Shioya et al., 2010) which are ethically unacceptable in life. Thus, a better understanding of the involvement of miRNAs in ALS is required. On the other hand, early diagnosis and management in specialized ALS clinics providing multidisciplinary patient care has been shown to positively impact quality of life and prolong survival of ALS patients. However, delay in diagnosis for ALS has been widely reported due to its significant overlap of clinical manifestations with some clinically alike conditions at the early stage of ALS (Andersen et al., 2012). Therefore, exploring the robust biomarkers is essential for early diagnosis of ALS. Unlike other biomarkers, miRNAs can be easily secreted into the extracellular space from neurons and other CNS cells. Also, miRNAs are isolated and quantified from a variety of biofluids, allowing miRNAs as ideal potential biomarkers for ALS. However, in the studies mentioned above, the samples were mostly obtained at the relatively end stage of the disease (De Felice et al., 2012; Ishtiaq et al., 2014).

Considering the limited stability of specific brain-enriched miRNAs due to the long interval for tissue samples to be obtained, and the difficulties of obtaining samples from brain, spinal cord or muscle (Sethi and Lukiw, 2009), it seems to be of great advantage to test for biomarkers presented in peripheral blood for diseases. Further evidence suggest that peripheral blood gene and miRNAs expression could be used as indicators in neurological diseases, such as Parkinson's disease (PD) (Serafin et al., 2015), Huntington's disease (Borovecki et al., 2005), and Alzheimer's disease (Cheng et al., 2014). Moreover, in order to avoid the effect of anticoagulation on the expression level of miRNAs (Hastings et al., 2012), we investigated potentially differential expression of miRNAs in leukocytes in SALS patients.

## Methods

This study was approved by the Ethics Committee of West China Hospital of Sichuan University, Chengdu, P.R. China, and written informed consent was obtained from all subjects before blood collection.

### Subjects

The peripheral blood from SALS, PD and healthy controls were obtained from the Department of Neurology, West China Hospital of Sichuan University between May 2011 and October 2012. Clinically definite SALS patients were diagnosed based on the EI Escorial revised criteria (Brooks et al., 2000) and PD patients were diagnosed according to the United Kingdom PD Society Brain Bank Clinical Diagnostic Criteria for PD (Hughes et al., 1992). Revised ALS-FRS (ALSFRS-R) was used to assess the disease severity as our previous study (Chen et al., 2014). To facilitate clinical interpretation of findings, ALSFRS-R scores were categorized into three stages of severity: mild or early (high, 37–48); moderate (medium, 25–36); and severe or late (low, 0–24) (Kimura et al., 2006). The microarray experiment included 5 SALS patients (3 males and 2 females) and 5 age- and sex-matched healthy controls. The mean disease duration of ALS patients was 15.19 ± 2.08 months (range from 12.32 to 17.85 months), the ALSFRS-R of them was 40.20 ± 1.92 (range from 38 to 43), and the mean age of SALS patients and controls was 50.75 ± 0.3 and 50.99 ± 0.5 years, respectively. Taqman Real Time-PCR experiments were performed on an independent group including 83 SALS patients, 24 PD patients and 61 healthy controls. All SALS patients were followed up every 3 months from the first registration.

### Leukocytes collection and RNA extraction

Approximately 5 ml of whole blood was collected in EDTA-containing tubes by venipuncture from all the participants before clinical treatment was started in the morning. Within 2 h of the blood collection, the peripheral blood mononuclear leukocytes (PBMCs) were separated via centrifuging with a Ficoll-Paque PLUS (GE Healthcare Life Sciences, #17-1440-02) according to the manufacturer's instructions. Then, the total RNA from PBMCs was isolated with a miRNeasy Mini Kit (Qiagen, #217004). NanoDrop ND-1000 Spectrophotometer (NanoDrop Technologies) was used to precisely measure the concentration of RNA samples. The integrity of the RNA extracts was measured using the Agilent Bioanalyzer 2100 (Agilent Technologies Inc.).

### miRNA expression profiling analysis

The microarray hybridization of miRNAs was performed by Capitalbio Corporation, China, using GeneChip miRNA 3.0 Array (Affymetrix) which contain a total of 1733 human sapiens mature miRNA and 1658 human sapiens pre-miRNA probesets that cover those miRNAs included in the Sanger miRBase v17 and miRBase miRNA (www.mirbase.org). A total of 130 ng RNA from each sample of SALS patients and healthy controls was used for each miRNA microarray. Affymetrix GeneChip miRNA Array Procedure including: FlashTag RNA Labeling, hybridization, Washing and Staining, scanning of the chips and analysis of the results was carried out following the protocols and equipment recommended by Affymetrix Inc. The software used for processing chips and the results was the Affymetrix GeneChip Command Console Software v3.0 (Affymetrix®). Data summarization, normalization, and quality control were used by the free miRNA QC Tool software (Affymetrix®), the detail information of which is shown in its protocol (http://media.affymetrix.com/support/downloads/manuals/mirna_qctool_user_manual.pdf). Briefly, the probe intensity values were background corrected, quantile-normalized, and the probeset-level expression signals were summarized with the robust multi-array average (RMA) method. The signal-to-noise ratio optimization method was used to estimate the differential expression for miRNAs, and the selection standard is as follow: it is defined as up-regulated when the *p* < 0.05 and the ratio>2 (patients to controls), and it is defined as down-regulated when the *p* < 0.05 and the ratio<0.5 (patients to controls; Seo et al., 2004).

### Validation analysis by quantitative Real-Time PCR (qRT-PCT)

Selected miRNAs were validated in the validation phase (83 SALS, 24 PD, and 61 healthy controls) by using SYBR green quantitative RT–PCR assay (Qiagen, Germany) (hsa-miR-34a, hsa-miR-100, hsa-miR-193b, hsa-miR-4485, hsa-miR-3690, hsa-miR-124, hsa-miR-183, hsa-miR-3935, hsa-miR-451, hsa-miR-4538, and hsa-miR-4701). The process of quantification included a two-step reaction: reverse transcription (RT) and PCR. Each RT reaction consisted of 2 μl total RNA, 2 μl miScript Reverse Transcriptase Mix (Qiagen, Germany), 2 μl miScript Nucleics Mix (Qiagen, Germany), and 4 μl miScript HiSpec Buffer (Qiagen, Germany), in a total volume of 20 μl. Reactions were performed in a C1000 Thermal Cycler (Bio-Rad, USA) for 60 min at 37°C, followed by heat-inactivation of RT for 5 min at 95°C. The 20 μl RT reaction mix was then diluted to 200 μl in nuclease-free water and held at −20°C.

qRT-PCR was performed using Bio-Rad CFX96 Real-Time PCR System (Bio-Rad, USA) with 20 μl PCR reaction mixture that included 2 μl cDNA, 10 μl QuantiTect SYBR Green PCR Master Mix (Qiagen, Germany), 2 μl miScript Universal Primer (Qiagen, Germany), 2 μl miScript Primer Assay (Qiagen, Germany), and 4 μl nuclease-free water. Reactions were incubated in a 96-well optical plate at 95°C for 15 min, followed by 40 cycles of 95°C for 15 s, 55°C for 30 s and 70°C for 30 s. Each sample was run in triplicate for analysis. At the end of the PCR cycles, melting curve analyses were performed to validate the specific generation of the expected PCR product. The expression changes of the miRNAs were normalized to RNU6, and the difference was calculated by the ΔΔCt method. The control group in each comparison was used as a calibrator (ΔΔCt = 0, 2^−ΔΔCt^ = 1). Amplification efficiency for each miRNA was assessment by technical duplicates method on a Bio-Rad Real-Time PCR System.

### Target prediction and gene ontology (GO) analysis

The target genes for each significantly differentially regulated miRNA were predicted by searching them on public databases endowed with prediction algorithms, such as TargetScan (http://targetscan.org), PicTar (http://pictar.mdc-berlin.de), RNA22 (https://cm.jefferson.edu/rna22/Interactive/), PITA (http://genie.weizmann.ac.il/pubs/mir07/mir07_data.html), and Miranda (http://www.mirbase.org).

Gene Ontology analysis was applied to analyze the main function of the differential expression genes according to the Gene Ontology which is the key functional classification of NCBI, which can organize genes into hierarchical categories and uncover the gene regulatory network on the basis of biological process and molecular function.

### Statistical analysis

Data were expressed as mean ± standard deviation (SD) or proportions. Student's *t*-test and Fisher's exact test were used to determine the difference of clinical characteristics between two groups. The differential expression levels of miRNAs were compared using the Mann–Whitney *U*-test, and the p values were corrected for multiple testing using the Benjamini–Hochberg false discovery rate (FDR) method. Receiver operating characteristics (ROC) curves and the area under the ROC curve (AUC) were established to evaluate the diagnostic value of miRNAs for differentiating patients from controls. An AUC of 0.5 indicates classifications assigned by chance. Based on ROC analysis, the best statistical cut-off values of miRNAs were calculated, and the sensitivity and specificity for selected cut-off points were then assessed. MedCalc (version 11.4.2.0; MedCalc, Mariakerke, Belgium) software was used to perform the ROC and regression analysis. All statistical analysis was performed using Graphpad Prism 5.01 (Graphpad Software Inc., San Diego, CA, USA). *P*-values of <0.05(two-tailed) were considered statistically significant.

## Results

### Clinical characteristics

The clinical characteristics of participants were summarized in Table 1. The mean age of SALS, PD and controls was 55.92 ± 7.38, 58.51 ± 5.60, and 54.20 ± 12.78 years, respectively. The mean disease duration of SALS and PD was 17.33 ± 13.68 and 28.92 ± 12.84 months, respectively. There were no significant differences in the distribution of sex among SALS, PD, and healthy controls. During the time when we analyzed the data of miRNAs expression, 58 patients died and the mean survival time of ALS patients was 28.44 ± 15.96 months.

**Table 1:** **General characteristic of the study subjects for validation study**.

	**SALS**	**PD**	**HCs**
Cases, n	83	24	61
Sex, female (%)	37 (44.58%)	10 (41.66%)	27 (44.26%)
Mean age (mean ± SD, years)	55.92 ± 7.38	58.51 ± 5.60	54.20 ± 12.78
Mean onset age (mean ± SD, years)	54.49 ± 6.35	56.67 ± 4.87	−
Disease duration^*^ (mean ± SD, months)	17.33 ± 13.68	28.92 ± 12.84	−
Mean ALSFRS-R	38.50 ± 6.22	−	−
**INITIAL SYMPTOMS**			
Bulbar	16	−	−
Upper limb	47	−	−
Lower limb	20	−	−
**ONSET AGE**			
≤40 years	12	−	−
>40 years	71	−	−
Disease duration^#^ (mean ± SD, months)	28.44 ± 15.96	−	−

* *Mean disease duration from onset to collection in all SALS patients*.

# *Mean disease duration from onset to death in 58 SALS*.

### Identification of differential miRNA profiling between ALS patients and healthy controls

**Figure 1:**
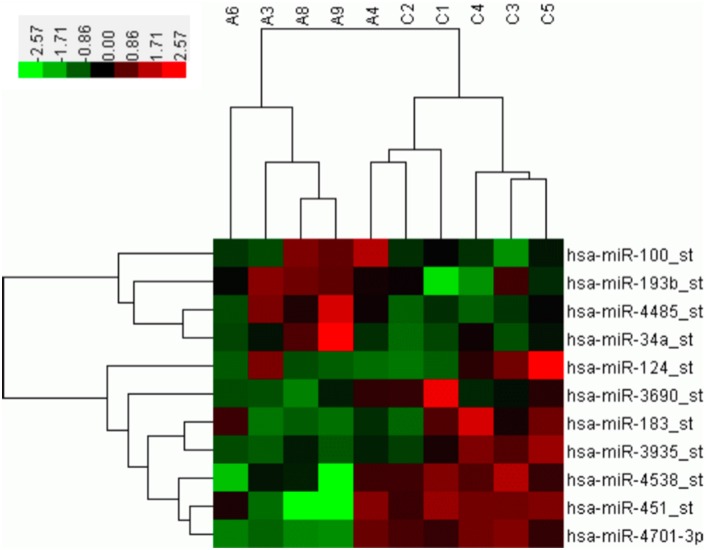
**Hierarchical clustering analysis between 5 SALS patients and 5 healthy controls based on differentially expressed miRNAs**. The horizontal rows represent the 11 differentially expressed microRNAs, and the columns represent the 10 samples, 5 from the SALS (A) and 5 from the healthy controls (C). Red indicates relative overexpression, and green indicates relative underexpression.

A microarray containing probes for 3391 human miRNAs (1733 mature and 1658 pre-miRNAs) was initially used to screen the differential expression levels of miRNAs between the SALS patients and healthy controls. Supplementary Figure 1 showed the hierarchical clustering of differential expressed miRNAs between SALS and controls. Four miRNAs, including hsa-miR-34a, hsa-miR-100, hsa-miR-193b, hsa-miR-4485, with significantly higher expression levels were identified in the SALS group compared with those in the healthy control group (mean ratio = 2.11−3.12, *p* < 0.05, Table 2, Figure 1). Seven miRNAs with significantly lower expression levels, including hsa-miR-3690, hsa-miR-124, hsa-miR-183, hsa-miR-3935, hsa-miR-451, hsa-miR-4538, and hsa-miR-4701, were identified in the SALS group compared with HCs (mean ratio = 0.35–0.43, *p* < 0.05, Table 2, Figure 1).

**Table 2:** **Differentially expression miRNAs identified by microarray analysis in leukocytes between SALS and controls**.

**Differentially expression type**	**microRNA name**	**Mean ratio**
Up-regulated	hsa-miR-193b	2.1165
	hsa-miR-34a	3.1282
	hsa-miR-100	2.3189
	hsa-miR-4485	2.6155
Down-regulated	hsa-miR-3690	0.4276
	hsa-miR-124	0.3538
	hsa-miR-183	0.3946
	hsa-miR-3935	0.3999
	hsa-miR-451	0.4126
	hsa-miR-4538	0.3603
	hsa-miR-4701	0.4249

### Validation analysis by quantitative Real-Time PCR

**Figure 2:**
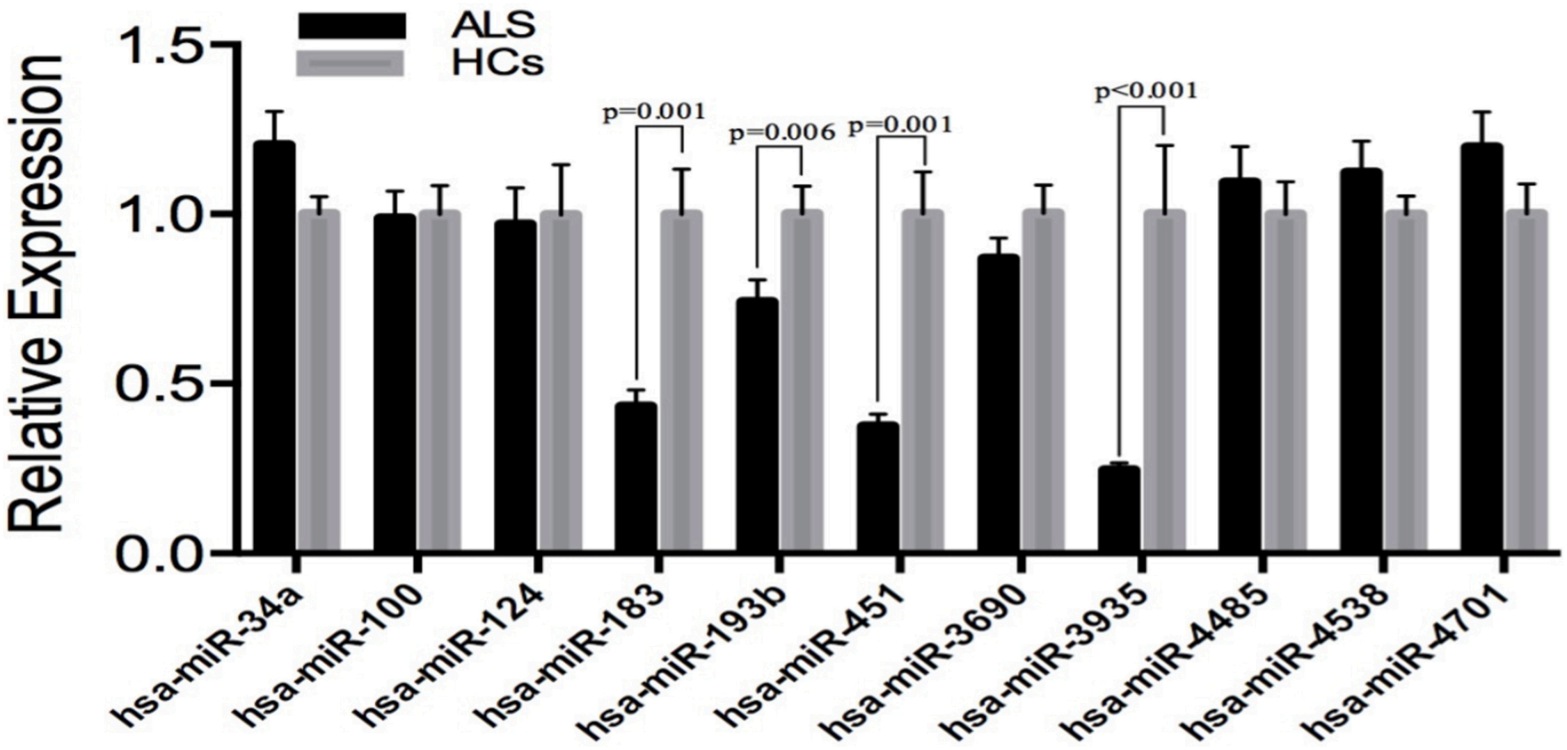
**Validation of miRNAs expression by qRT-PCR analysis in leukocytes samples from SALS (***n*** = 83) and HCs (***n*** = 61)**. Data are represented by bar chart. The *P*-values were calculated by the Mann-Whitney *U*-test. The relative expression of miRNAs in HCs was normalized.

In order to avoid the false-positive results obtaining from microarray, we performed additional real-time PCR for all of the 11 identified different expressed miRNAs in an independent cohort, including 83 SALS and 61 HCs to validate. The expression patterns of these miRNAs were analyzed using quantitative real-time PCR (qRT-PCR). The significant differences in the expression levels of four miRNAs (hsa-miR-183, hsa-miR-193b, hsa-miR-451, and hsa-miR-3935) were confirmed between the SALS patients and HCs (Figure 2). It is noteworthy that the hsa-miR-193b showed opposite direction of relative expression with microarray analysis in the validation stage.

There were no significant differences in the expression levels of the four miRNAs between patients with spinal-onset and bulbar-onset, between patients with disease duration within 1 year and within 1–2 years, between patients with early onset (≤40 years) and late onset (>40 years), and between patients with different sex. In addition, no correlation was found between the expression levels of the four miRNAs and disease duration from onset to death (data not shown).

**Figure 3:**
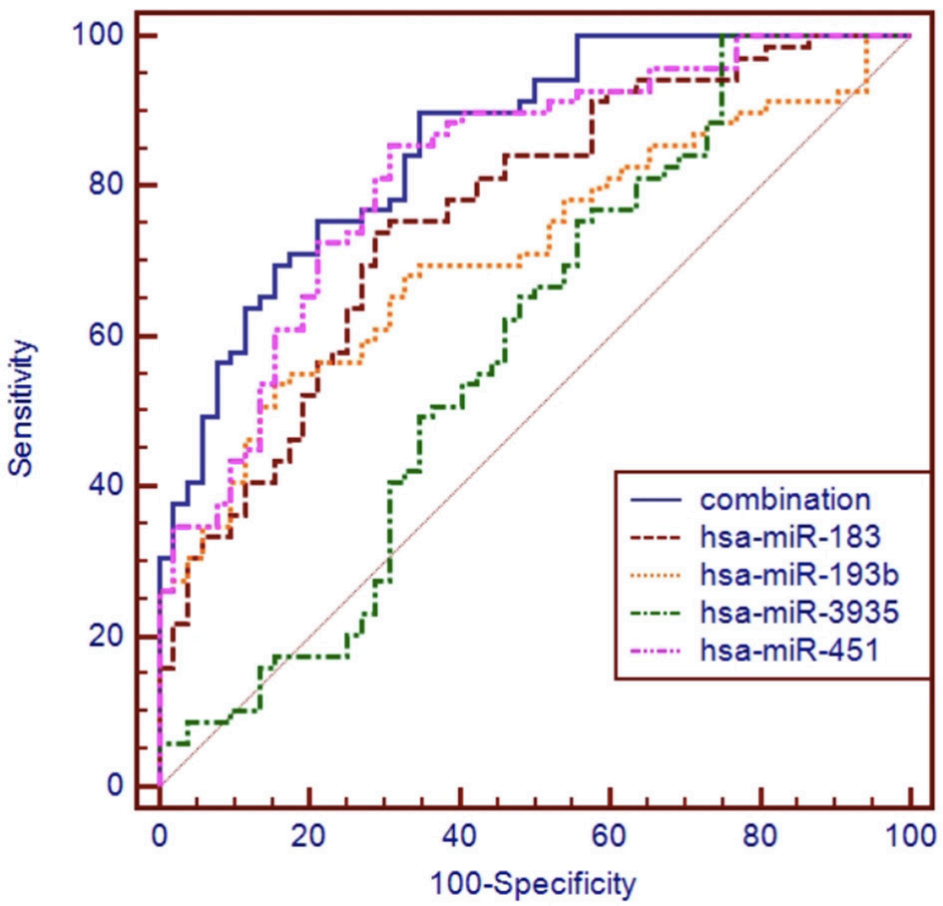
**ROC curve analysis using differential expressive miRNAs for discriminating SALS**. ROC curve analyses revealed that the plasma levels of hsa-miR-183, hsa-miR-193b, hsa-miR-451, and hsa-miR-3935 were useful biomarkers for differentiating SALS and HCs, with AUC values of 0.763, 0.713, 0.820, and 0.590, respectively. When the four miRNAs were combined by logistic regression model, the AUC values for differentiating SALS and HCs were 0.857.

The ROC curve analyses revealed that the levels of hsa-miR-183, hsa-miR-193b, hsa-miR-451, and hsa-miR-3935 were useful biomarkers for the diagnosis of ALS, with AUC values of 0.763 [95%CI: 0.677–0.835], 0.713 [95%CI: 0.624–0.792], 0.820 [95%CI: 0.740–0.884], and 0.590 [95%CI: 0.497–0.679], respectively. Among the four miRNAs, hsa-miR-193b had the highest specificity with the cut-off value of 0.7675, and hsa-miR-3935 had the highest sensitivity when its cut-off value is 4.2734 (Table 3). The four miRNAs were combined as a panel of miRNAs by the logit model, which was used to construct the ROC curve as follow, logit (*p* = SALS) = 3.152 − 0.708 × hsa-miR-193b − 1.517 × hsa-miR-183 − 0.147 × has-miR-3935 − 3.331 × hsa-miR-451. The calculated AUC for the miRNA panel was 0.857 (95%CI: 0.782–0.914, sensitivity = 89.9%, specificity = 65.4%, Table 3, Figure 3).

**Table 3:** **miRNAs profile and diagnostic performance in training dataset**.

**micRNAs**	***p*-valve**	**AUC**	**95%CI**	**Z statistic**	**Criterion**	**Sensitivity (%)**	**Specificity (%)**
hsa-miR-193b	0.006	0.713	0.624–0.792	4.592	≤0.7675	53.6	84.6
hsa-miR-183	0.001	0.763	0.677–0.835	6.043	≤0.3727	73.9	71.2
hsa-miR-3935	<0.001	0.590	0.497–0.679	1.646	≤4.2734	100	25.0
hsa-miR-451	0.001	0.820	0.740–0.884	8.361	≤0.2557	85.5	69.2
Combination^*^		0.857	0.782–0.914	10.818	> −0.0929	89.9	65.4

* *Logit (p = SALS) = 3.152 − 0.708 × hsa-miR-193b − 1.517 × hsa-miR-183 − 0.147 × has-miR-3935 − 3.331 × hsa-miR-451*.

In order to assess whether the four different expression miRNAs were specific in ALS, the expression levels of the four miRNAs were tested in 24 PD patients. The expression of hsa-miRNA-183 showed no difference between PD patients and HCs, but was significantly lower in SALS patients than that in PD patients. The expression of hsa-miR-451 and hsa-miR-3935 were significantly lower in PD patients than those in SALS and controls. There was no significant difference in the expression of hsa-miR-193b between PD patients and HCs, and between SALS patients and PD patients (Table 4).

**Table 4:** **Different expression of all four miRNAs in SALS patients, PD patients, and healthy control subjects**.

**miRNAs**	**SALS**	**PD**	**HCs**	***p*-value^a^**	***p*-value^b^**	***p*-value^c^**
hsa-miR-183	0.338 ± 0.2370	1.5258 ± 1.0873	0.7406 ± 0.7409	<0.0001	0.001	0.3887
hsa-miR-193b	1.1993 ± 0.7731	1.3179 ± 0.8240	1.6722 ± 1.1496	0.5533	0.006	0.184
hsa-miR-451	0.193 ± 0.1583	0.1161 ± 0.0746	0.5935 ± 0.5139	0.0084	<0.0001	<0.0001
hsa-miR-3935	1.5258 ± 1.0873	0.8964 ± 0.3793	6.5546 ± 8.7273	0.0008	0.001	<0.0001

a *Different expression between SALS and PD patients*.

b *Different expression between SALS patients and healthy control subjects*.

c *Different expression between PD patients and healthy control subjects*.

### Target prediction and gene ontology (GO) analysis

**Figure 4:**
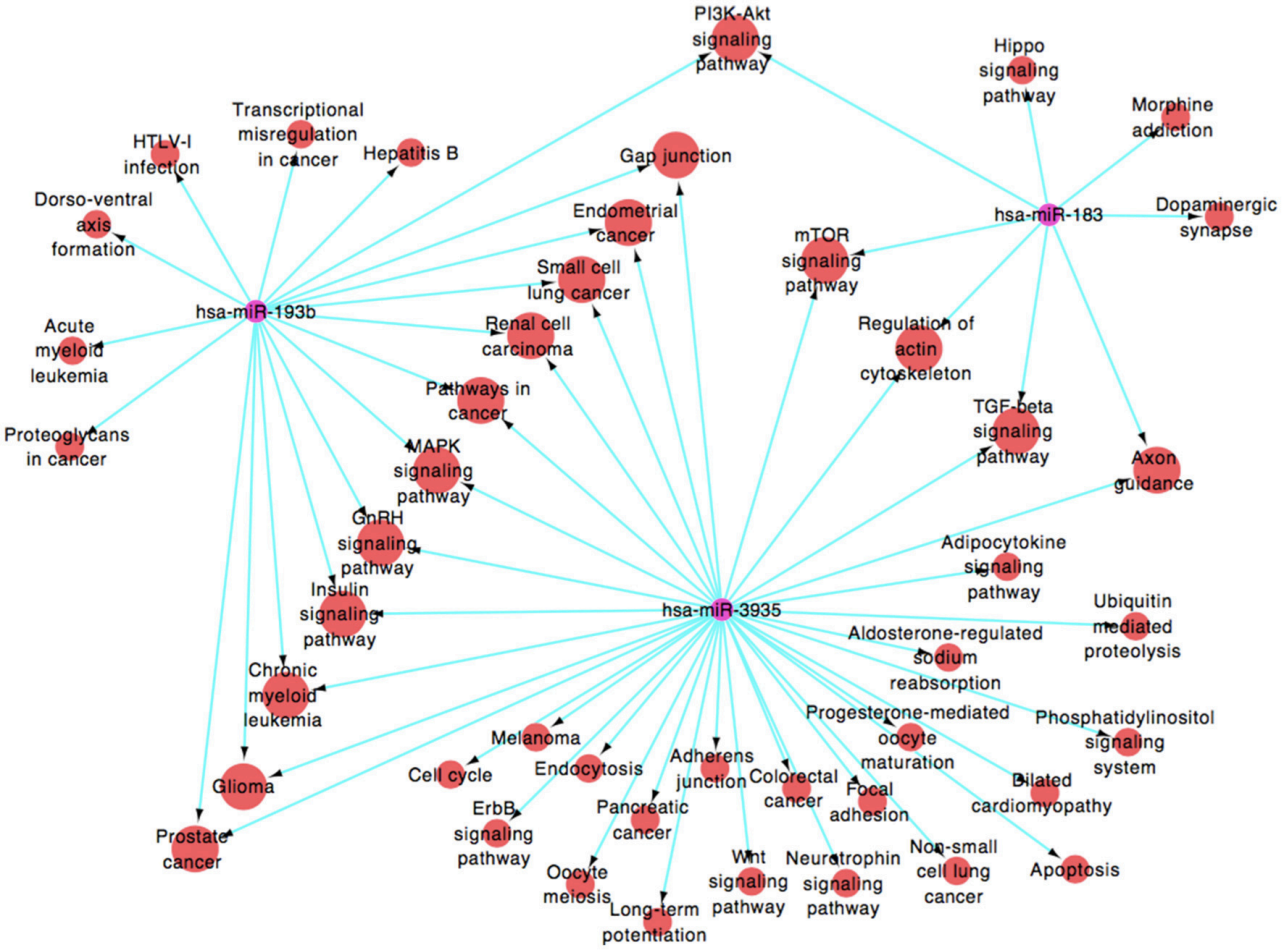
**miRNA-pathway signal-net analyses**.

By public miRNA target databases, 182 genes that were predicted to be target genes of at least 2 miRNAs in these four miRNAs (Supplementary Figure 1 and Supplementary Table 1 [miRNAs-target genes]) were obtained. A functional network (Figure 4 and Supplementary Table 1 [miRNAs-pathways]), based on Gene Ontology and KEGG database, was constructed to visualize the connections of hsa-miR-183, hsa-miR-193b, hsa-miR-3935, and their predicted targets (hsa-miR-451, which target genes were less and failed to construct the pathway net, was rule out). Forty-five pathways were constructed, including 16 pathways linked to the two miRNAs, among which PI3K-Akt signaling pathway (map04151), mTOR signaling pathway (map04150), regulation of actin cytoskeleton (map04810), axon guidance (map04360), MAPK signaling pathway (map04010), glioma (map05214), and gap junction (map04540), were associated with the pathogenic mechanism with neurodegenerative diseases.

## Discussion

In this study, we were the first to report a comprehensive array-based miRNA expression profile in the peripheral blood of SALS patients from Han Chinese. We observed four down-regulated miRNAs in SALS contrasted with that in healthy controls and found that hsa-miRNA-183 might be a specific biomarker for SALS.

According to the results of our previous study (Yang et al., 2011), the mean survival time of SALS patients was 32.44 ± 19.63 months and the median survival time was 29.67 months. However, the survival time of ALS patients is much shorter in our cohort than that in Caucasian populations (Martínez et al., 2011). Besides the disparity of the medical condition, delay in diagnosis is an important factor affecting the mean survival time. Previous retrospective studies found a delay from symptom onset to diagnosis ranges from 8 to 15.6 months (Cellura et al., 2012). Another study reported that more than half of ALS patients received an alternative diagnosis and each patient saw an average of three different physicians before ALS diagnosis was confirmed (Paganoni et al., 2014). Moreover, time to diagnosis are longer for patients with limb-onset, older age, or sporadic form (Paganoni et al., 2014). In China, fewer neurologists were available for the same population-based community comparing with that in the developed countries. Therefore, discovery of an accurate and sensitive biomarker is of utmost importance in current ALS research. Biomarkers in blood are the primary consideration due to its advantages of non-invasive, easy handling and multiple testing at a low cost. Further, the steady miRNAs is a more promising potential biomarker than cytokines and growth factors in plasma and serum.

MiRNAs, as a key regulator, was first reported by Williams et al. That study showed that miR-206 slowed ALS progression and promoted regeneration of neuromuscular synapses in mice model (Williams et al., 2009). Ever since, numerous studies about miRNAs in ALS have been conducted, the samples were obtained from cultured cells, animal model and ALS patients, and the tissues included blood, cerebral spinal fluid (CSF), muscle, spinal cord, and brain (Cloutier et al., 2015). However, as a biomarker, miR-206 was reproduced by only one study, which reported that miR-206 was up regulated in serum from SOD1 ALS mice and ALS patients (Toivonen et al., 2014). Except for this study, few miRNAs were reproduced in the following studies whether microarray or qRT-PCR based. Possible explanations for these different results included detection methods, sample size, resource of tissue, disease course, familial or sporadic form, and ethnic origin. Some studies only used microarray method without verifying the facticity; some studies only applied qRT-PCR method without discovering the novel miRNAs; while other studies recruited patients or controls less than 10, which easily resulted in false positive or false negative findings. Furthermore, miRNAs expressions may varied at different stages as the disease progresses, the different expression of miRNAs in fALS patients could not be reproduced in SALS patients probably due to the specific defective genes in fALS, and a more heterogeneous of miRNAs in SALS compared with fALS were identified (Freischmidt et al., 2015). Thus, the expression of miRNAs might be regulated by some upstream genes, the epigenetic modifications, which were by mediated in different genetic background. In the present study, using the methods of combining microarray and qRT-PCR, we first detected miRNAs in Chinese SALS population. These miRNAs, including hsa-miR-183, hsa-miR-193b, hsa-miR-451, and hsa-miR-3935, were down regulated in SALS patients relative to healthy controls, with AUC values of 0.69, 0.62, 0.81, and 0.57, respectively. Interestingly, in the subgroup analysis, no significant differences in the expression levels of these four miRNAs were found, according to clinical presentations of ALS, such as site of onset (spinal or bulbar), age of onset (early or late), disease duration (within one year and within 1–2 years), and gender. Our finding may indicate that these miRNAs could provide a new promising opportunity for detection of SALS. Combining the expression of these four miRNAs in PD, the results suggested that hsa-miRNA-183 might be a specific biomarker for SALS, whereas hsa-miR-451 and hsa-miR-3935 might be common biomarkers linked to neurodegenerative diseases, such as ALS and PD. However, these need to be confirmed in further related studies with other neurodegenerative diseases and a larger number of participants.

There is growing evidence suggesting that specific miRNAs are involved in the development and progression of ALS through the regulation of broad signaling pathway, including inflammation (Butovsky et al., 2012; Parisi et al., 2013), neurofilament (Campos-Melo et al., 2013; Ishtiaq et al., 2014), mitochondrial function (Russell et al., 2012), endoplasmic reticulum (ER) stress (Nolan et al., 2014) and others complex network of miRNAs (Shinde et al., 2013). The miR-29a-special antagomir, demonstrated increased lifespan in male SOD1^G93A^ mice by reducing ER stress (Nolan et al., 2014); Inhibition of miR-23a ameliorates skeletal muscle mitochondrial function in ALS by increasing the PGC-1α signaling, which was involved in mitochondrial biogenesis and function (Russell et al., 2012); the anti-miR-155 significantly extends survival and disease duration in the *SOD1*^G93A^ mouse model (Koval et al., 2013), and the miR-206 delays ALS progression and promotes regeneration of neuromuscular synapses in mice partly through fibroblast growth factor and histone deactylase 4 signaling pathways (Williams et al., 2009). In the present study, we observed significantly lower expression of hsa-miR-183, hsa-miR-193b, hsa-miR-451, and hsa-miR-3935 in SALS patients compared with controls. By target prediction and GO analysis, we found low levels of these miRNAs may ease post-transcriptional suppression of their up-regulated mRNA targets related to several neurodegenerative signaling pathway, including PI3K-Akt signaling pathway (hsa-miR-183 andhsa-miR-193b), mTOR signaling pathway (hsa-miR-193b and hsa-miR-3935), regulation of actin cytoskeleton (hsa-miR-193b and hsa-miR-3935), axon guidance (hsa-miR-193b and hsa-miR-3935), MAPK signaling pathway (hsa-miR-183 andhsa-miR-3935) and so on. The differential expression of miR-183 was found in several cancers, including prostate cancer (Yuan et al., 2015), gastric cancer (Cao et al., 2014), breast cancer (Lowery et al., 2010), which may be involved in cell proliferation, migration and invasion (Zhu et al., 2012; Lu et al., 2015). The miR-183-96-182 cluster regulates multiple biological programs that converge to support the maintenance and metastatic potential of medulloblastoma coupled to the PI3K/AKT/mTOR pathway (Weeraratne et al., 2012). Previous studies also found that survival motor neuron (SMN) gene regulates axonal local translation via miR-183/mTOR pathway, which contributed to spinal muscular atrophy pathology (Kye et al., 2014). Thus, dysregulation of miR-183 may be involved in progressive loss of motor neurons in ALS. Similarly, the miR-193b was also reported to modulate cell proliferation (Chen et al., 2010; Gao et al., 2011), migration, and invasion (Hu et al., 2012), and downregulation of miR-193b contributes to enhanced tumor progression and invasion through its target gene-neurofibromin 1(NF1) (Li et al., 2009; Lenarduzzi et al., 2013). The NF1 is required for appropriate granule neuron progenitor expansion and migration in cerebellar development through regulation of RAS/ERK signaling (Sanchez-Ortiz et al., 2014). Moreover, previous study found the miR-193b may take part in the development of AD and is a potential blood-based biomarker of mild cognitive impairment and AD (Liu et al., 2014). However, the accurate mechanisms of the miR-193b causing neurodegeneration remained unknown. Previous studies found that the miR-451, which was differentially expressed in several diseases, such as multiple myeloma (Du et al., 2015), epithelial ovarian cancer (Ling et al., 2015), and central nervous system malignancies (Drusco et al., 2015), could represses cell proliferation and invasion (Zeng et al., 2014). In SH-SY5Y neuron-like cells, downregulation of miR-451 had neurotrophic, neuroprotective, anti-Oxidant, and anti-apoptotic effects (Alural et al., 2014). Interestingly, the down-regulation of hsa-miR-451 was also found in leukocytes from Italian ALS patients (De Felice et al., 2012). In the present study, we observed down-regulation of hsa-miR-451 in our SALS and PD patients, suggesting that the miRNA might be a biomarker for neurodegenerative diseases, which was not specific to SALS. However, little is known about the function and role of miR-3935. In the current study, the down-regulation of miR-3935 was found not only in SALS but also in PD compared with healthy controls, indicating that miR-3935 miRNA might be related to neurodegeneration.

However, we must consider some limitations of the current study. First, only one sample per patient was test in microarray analysis study, which was one of confounding factors resulting in the relatively high variability of miRNAs expression, although the quality of each sample of subjects were strictly tested. Second, for the potential candidate biomarker study, longitudinal data providing is important. Regrettably, the blood sample for each subject was collected only once in the current study. The expression profiling of ALS during disease progression should be tested in the future studies. Thirdly, only PD patients besides ALS were enrolled in the validation stage analysis for comparation, therefore other neurodegenerative disorders such as AD and FTD and so on should be compared. And whether alteration of these miRNAs in the CNS should be tested in the future studies.

In summary, our pilot study showed that peripheral blood leukocytes miR-183, miR-193b, miR-451, and miR-3935 are down regulated in SALS, which raises the potential clinical utility of leukocytes miRNA profiling in SALS diagnosis. Although, such approach is technically challenging, we believe that the leukocytes miRNAs could be a promising diagnostic tool for SALS. Further investigations are required to figure out the underlying mechanisms of how the change of miRNAs level in leukocytes reflect the pathology of motor neuron, as well as their biological functions.

## Author contributions

HS planned the study. YC, QW, XC, CL, BC, RO, and HS collected and analyzed clinical data, and made patient follow-ups. YC conducted the molecular studies. SH interpreted data. YC wrote the article, and HS edited the paper.

### Conflict of interest statement

The authors declare that the research was conducted in the absence of any commercial or financial relationships that could be construed as a potential conflict of interest.
