# Supplementary material for: Aberration of miRNAs Expression in Leukocytes from Sporadic Amyotrophic Lateral Sclerosis
Source: Front Mol Neurosci. 2016 Aug 17;9:69. doi: 10.3389/fnmol.2016.00069 (PMC4987348; doi:10.3389/fnmol.2016.00069)

Supplementary figure 1 Target genes of these 4 miRNAs

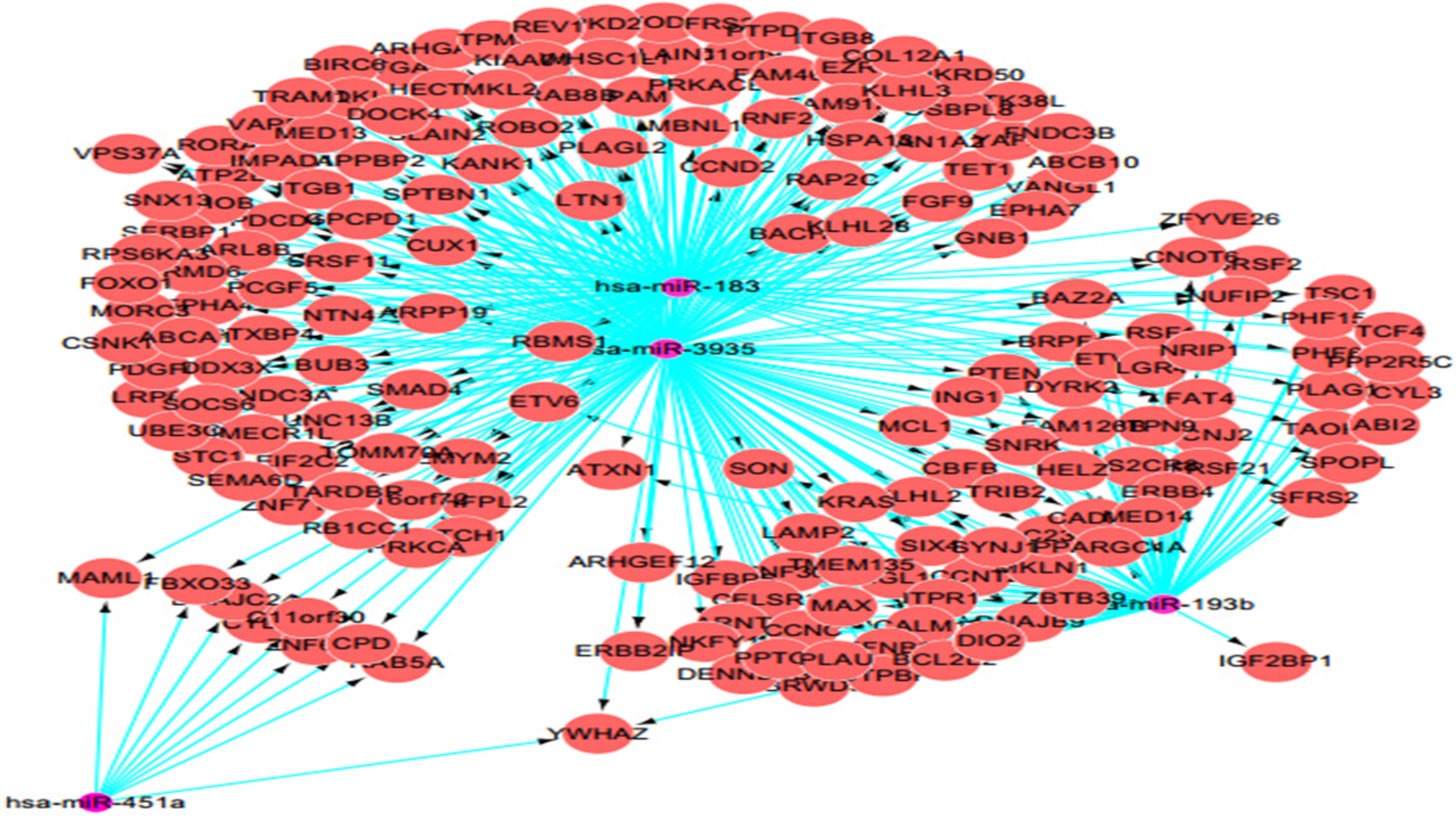

Supplement: Supplementary file 2 [file Image1.PDF]
